# G protein-coupled estrogen receptor 1 regulates renal endothelin-1 signaling system in a sex-specific manner

**DOI:** 10.3389/fphys.2023.1086973

**Published:** 2023-01-17

**Authors:** Ginger L. Guthrie, Rawan N. Almutlaq, Sho Sugahara, Maryam K. Butt, Craig R. Brooks, David M. Pollock, Eman Y. Gohar

**Affiliations:** ^1^ Division of Nephrology, Department of Medicine, University of Alabama at Birmingham, Birmingham, AL, United States; ^2^ Division of Nephrology and Hypertension, Vanderbilt University Medical Center, Nashville, TN, United States

**Keywords:** GPR30, sex differences, kidney medulla, sodium handling, blood pressure

## Abstract

Demographic studies reveal lower prevalence of hypertension among premenopausal females compared to age-matched males. The kidney plays a central role in the maintenance of sodium (Na^+^) homeostasis and consequently blood pressure. Renal endothelin-1 (ET-1) is a pro-natriuretic peptide that contributes to sex differences in blood pressure regulation and Na^+^ homeostasis. We recently showed that activation of renal medullary G protein-coupled estrogen receptor 1 (GPER1) promotes ET-1-dependent natriuresis in female, but not male, rats. We hypothesized that GPER1 upregulates the renal ET-1 signaling system in females, but not males. To test our hypothesis, we determined the effect of GPER1 deletion on ET-1 and its downstream effectors in the renal cortex, outer and inner medulla obtained from 12–16-week-old female and male mice. GPER1 knockout (KO) mice and wildtype (WT) littermates were implanted with telemetry transmitters for blood pressure assessment, and we used metabolic cages to determine urinary Na^+^ excretion. GPER1 deletion did not significantly affect 24-h mean arterial pressure (MAP) nor urinary Na^+^ excretion. However, GPER1 deletion decreased urinary ET-1 excretion in females but not males. Of note, female WT mice had greater urinary ET-1 excretion than male WT littermates, whereas no sex differences were observed in GPER1 KO mice. GPER1 deletion increased inner medullary ET-1 peptide content in both sexes but increased outer medullary ET-1 content in females only. Cortical ET-1 content increased in response to GPER1 deletion in both sexes. Furthermore, GPER1 deletion notably increased inner medullary ET receptor A (ET_A_) and decreased outer medullary ET receptor B (ET_B_) mRNA expression in male, but not female, mice. We conclude that GPER1 is required for greater ET-1 excretion in females. Our data suggest that GPER1 is an upstream regulator of renal medullary ET-1 production and ET receptor expression in a sex-specific manner. Overall, our study identifies the role of GPER1 as a sex-specific upstream regulator of the renal ET-1 system.

## Introduction

Multiple demographic reports demonstrate gender differences in the prevalence of cardiovascular diseases including hypertension (Burt et al., 1995; Lloyd-Jones et al., 2009). Ambulatory 24 h blood pressure studies report lower blood pressure in women compared to age-matched men, until menopausal age (Staessen et al., 1990; Khoury et al., 1992; Wiinberg et al., 1995), suggesting a protective role for ovarian hormones. Particularly, estradiol administration has been shown to elicit blood pressure lowering effects in various animal models (Magness et al., 1993; Williams et al., 1994; Davis et al., 2019; Đogo et al., 2021). Importantly, the kidney plays a central role in the maintenance of sodium (Na^+^) homeostasis and consequently blood pressure. Evidence suggests that estrogen signaling elicits renal protective effects in various injury models (Takaoka et al., 2002; Satake et al., 2008; Hutchens et al., 2010; Hutchens et al., 2012; Gohar et al., 2021). However, the interactions between estrogen signaling and blood pressure regulatory pathways in the kidney are not fully understood. Further investigating the interactions between estrogen signaling and Na^+^ homeostasis regulatory pathways may identify a therapeutic target for hypertension treatment.

The seven transmembrane G protein-coupled estrogen receptor 1 (GPER1, formerly called GPR30) is involved in mediating estrogen signaling (Carmeci et al., 1997). GPER1 has been shown to induce non-genomic effects *via* calcium mobilization and phosphatidylinositol 3,4,5-trisphosphate secondary messengers (Revankar et al., 2005; Boscaro et al., 2020). Activation of GPER1 by a selective agonist, G1, decreases blood pressure in ovariectomized (OVX) mRen2. Lewis rats (Lindsey et al., 2009), OVX Sprague Dawley (SD) rats (Gohar et al., 2020), and stimulates endothelium-dependent vasodilation in mesenteric vessels of female mRen2. Lewis rats (Lindsey et al., 2011) and in carotid arteries of male and female SD rats (Broughton et al., 2010). Furthermore, GPER1 activation has been shown to improve vascular function of the renal interlobular artery by increasing nitric oxide content (Chang et al., 2019). The expression level of GPER1 mRNA is sex-specific within cardiovascular and renal tissues (Hutson et al., 2019). Notably, GPER1 mRNA expression in female whole kidneys is two-fold greater than male kidneys from SD rats (Hutson et al., 2019). Our lab has shown that acute activation of renal medullary GPER1 by G1 promotes natriuresis and diuresis in female, but not male, SD rats (Gohar et al., 2020), providing evidence for GPER1 as a female-specific pro-natriuretic factor. Additionally, we have reported greater GPER1 mRNA expression and protein abundance in females compared to males in the outer, but not inner, medulla (Gohar et al., 2020).

The endothelin-1 (ET-1) signaling system is essential for the regulation of Na^+^ homeostasis (Kohan et al., 2011). Renal ET-1 signaling has been shown to regulate renal vascular tone (Yanagisawa et al., 1988; Evans et al., 2001; Just et al., 2004), tubular reabsorption (Edwards et al., 1990; Romano et al., 2000; Shi et al., 2007), and consequently blood pressure. ET-1 production is highest in the kidney compared to other cardiovascular organs (Kitamura et al., 1989), emphasizing the importance of ET-1 for the regulation of renal microcirculation and fine-tuning tubular Na^+^ reabsorption. ET-1 actions are mediated *via* stimulation of its downstream G protein-coupled receptors, ET receptor A (ET_A_) and ET receptor B (ET_B_) (Kitamura et al., 1999; Imamura et al., 2001). ET_A_ primarily regulates renal vascular tone (Takeda et al., 1992; Pollock and Opgenorth, 1994) while ET_B_ is the main facilitator of natriuresis (Gariepy et al., 2000; Nakano et al., 2008), and these receptors are predominately located in smooth muscle and collecting duct cells, respectively (Karet et al., 1993; Kuc and Davenport, 2004).

Sex-related discrepancies in renal ET-1 receptor expression level and function have been reported (Gohar et al., 2016a). Binding studies revealed that the inner medullary collecting duct (IMCD) from male SD rats has higher ET_A_ expression than females, whereas, the ET_B_ expression is similar between SD male and female rats (Jin et al., 2013). In ET_B_-deficient rats fed a high salt diet, females have a quicker natriuretic response than males (Johnston et al., 2016), and ET_B_-deficient female rats, but not males, elicits ET_A_-mediated natriuresis (Nakano and Pollock, 2009). OVX decreases ET_A_ and ET_B_ receptor expression in the renal cortex and increases their expression in the inner medulla of SD rat kidneys (Gohar et al., 2016b). However, estradiol treatment to OVX SD rats eliminates the OVX-induced increase in renal ET_A_ and ET_B_ receptor expression (Gohar et al., 2016b), pointing to estrogen as a regulator for renal ET receptor expression. Indeed, evidence suggest a potential crosstalk between GPER1 and ET-1 signaling pathways within the vascular (Meyer et al., 2012), cardiac (Goncalves et al., 2018), and renal systems (Gohar et al., 2020; Gohar and Pollock, 2021). Within the renal medulla of the female SD rat, G1-induced natriuresis is blunted by simultaneous ET_A_ and ET_B_ inhibition (Gohar et al., 2020; Gohar and Pollock, 2021), which provides supporting evidence that GPER1 is a female-specific pro-natriuretic factor due to its interactions with the renal ET-1 system, yet these signaling interactions are not completely understood.

To further investigate the interactions between GPER1 signaling and the renal ET-1 system, we utilized male and female GPER1 full-body knockout (KO) and wildtype (WT) mice. Blood pressure, heart rate, metabolic cage parameters, urinary and plasma electrolytes were measured. In addition, we determined genotypic and sex differences in ET-1 urinary excretion, peptide, and mRNA expression within the cortex, outer medulla, and inner medullary regions of the kidney. Immunohistology staining of ET-1 quantified and localized the renal cortical ET-1 content in terms of percent area stained. Furthermore, the effect of genetic deletion of GPER1 on the mRNA expression of renal cortical and medullary ET receptors was also determined. We hypothesized that GPER1 in females, but not males, upregulates the renal ET-1 signaling system as reflected by increased ET-1 urinary excretion, peptide, and mRNA expression.

## Methods

### Animal studies

**FIGURE 1:**
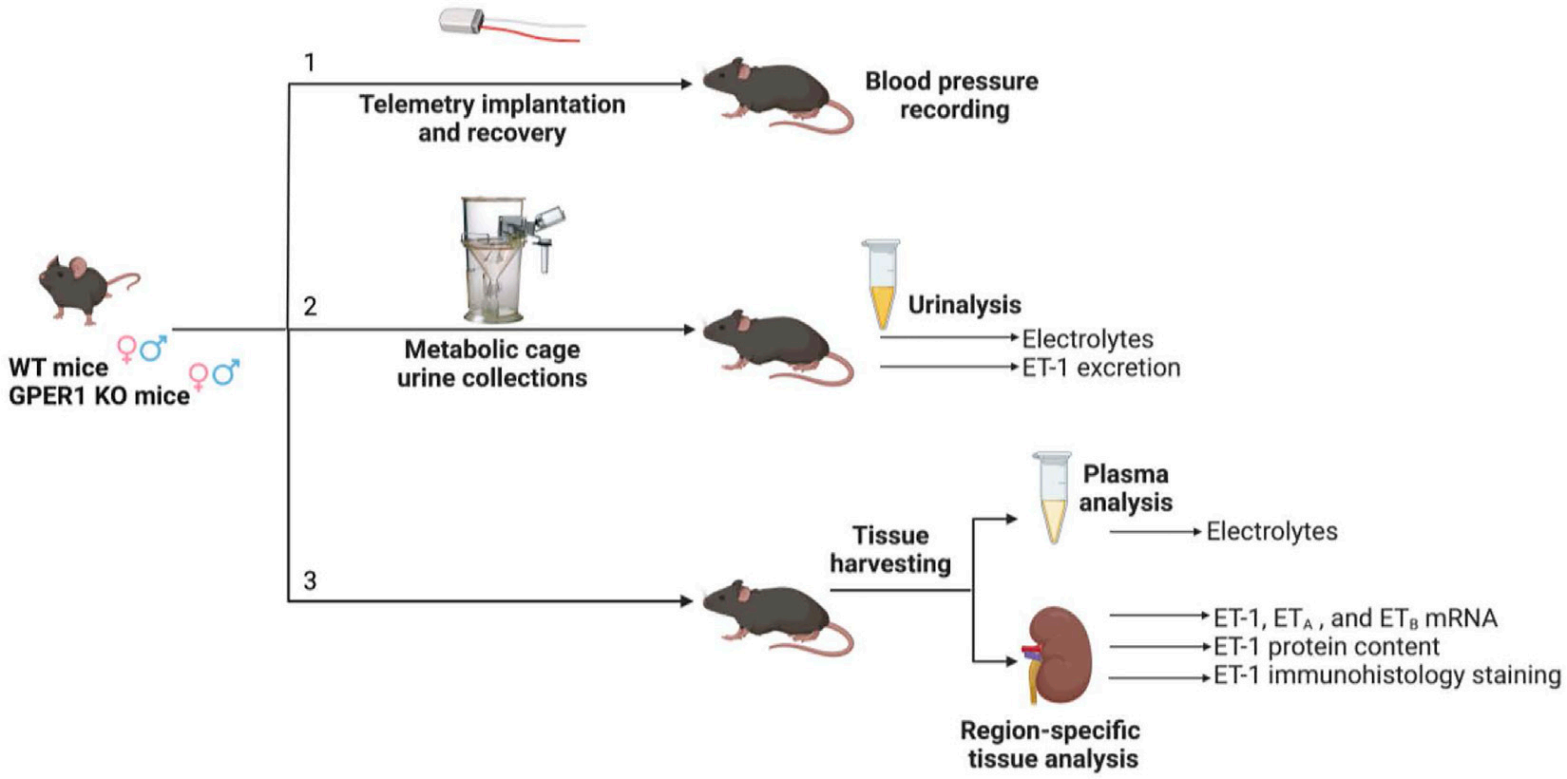
Experimental timeline. Three cohorts of mice separately underwent [1] telemetry experiments for blood pressure measurement, [2] metabolic cage experiments for 24 h urine collections, and [3] tissue harvesting. Each cohort included female and male GPER1 KO and WT mice. Abbreviations: ET-1, endothelin-1; ET_A_, endothelin receptor A; ET_B_, endothelin receptor B; GPER1 KO, G protein-coupled estrogen receptor 1 knockout; WT, wildtype. Created with BioRender.com.

All conducted animal procedures were approved by the University of Alabama at Birmingham (UAB) Institutional Animal Care and Use Committee and in accordance with ARRIVE Guidelines (Percie du Sert et al., 2020). Experiments were conducted using mice (females: WT: *n* = 28, GPER1 KO: *n* = 25; males: WT: *n* = 23, GPER1 KO: *n* = 30) obtained from our in-house colony. The generation of full body GPER1 KO mice has been previously described (Wang et al., 2008). Mice were genotyped to ensure proper GPER1 deletion. Throughout the experiments, animals were maintained in rooms with 12 h light/dark cycle (7 am-7 pm light) controlled for temperature (18°C–23°C), air pressure, and humidity with free access to a normal salt diet (TD.6208 0.49% NaCl; Envigo, Indianapolis, IN). Mice were randomly divided into three parallel groups for blood pressure measurement, metabolic cage studies, and tissue harvesting (Figure 1).

1) *Blood pressure measurement.* Mean arterial pressure (MAP) was measured using telemetry transmitters (PA-C10; Data Sciences International, Duluth, MN) as previously detailed (Zhang et al., 2020). At 11–15 weeks of age, mice (females: WT: *n* = 10, GPER1 KO: *n* = 10; males: WT: *n* = 6, GPER1 KO: *n* = 12) were anesthetized with 1.5% isoflurane, and telemetry transmitters were implanted into the right carotid artery. Sutures were used to close the muscle layer and the skin, each separately. Mice were allowed 14 days for postsurgical recovery before blood pressure measurement. Blood pressure was recorded for 10 s once every 10 min for five to seven consecutive days^1^.
2) *Metabolic cage study.* Another cohort of mice (females: WT: *n* = 12, GPER1 KO: *n* = 8; males: WT: *n* = 10, GPER1 KO: *n* = 12) were placed into metabolic cages at 11–15 weeks of age and allowed to acclimate for 2 days. Then, mice were monitored for food and water intake, and 24 h urine samples were collected between 7:30–8:30 a.m. for two consecutive days. Urine samples were centrifuged at 1,000 xg at 4°C for 5 min, aliquoted, and stored in −80°C.
3) *Tissue harvesting.* The third cohort of mice (females: WT: *n* = 6, GPER1 KO: *n* = 7; males: WT: *n* = 7, GPER1 KO: *n* = 6) with similar living conditions were euthanized and kidney tissues were harvested at 18–23 weeks of age between 7 and 9 am. One kidney was fixed for histological analysis. The other kidney was vertically bifurcated, sectioned into the cortex, outer and inner medulla, and snap frozen until stored in −80°C. Plasma was collected during this time using heparin to prevent coagulation and then stored in −80°C until analyzed.

### Assays

1) *Electrolyte measurements.* Urine and plasma Na^+^ and K^+^ concentrations were measured with an atomic absorbency spectrometer (iCE 3,000 series paired with a CETAC ASX‐520 AutoSampler; Thermo Fisher Scientific, Waltham, MA).
2) *Aldosterone measurement.* Urinary aldosterone levels were determined using a non-commercial immunoassay kit as previously described (Manolopoulou et al., 2008).
3) *ET-1 tissue extraction.* ET-1 was extracted from the renal cortex, outer, and inner medullary tissues following previously described protocol with some modifications (Sasser et al., 2002). Briefly, the renal cortex, outer, and inner medullary tissues were homogenized in 5, 10, and 50 volumes, respectively and relative to the mass of the tissue sample, of 1M acetic acid (984,303; Thermo Fisher Scientific) containing 10 ug/mL of pepstatin A protease inhibitor (78,436; Thermo Fisher Scientific). Samples were then centrifuged, and precipitates were resuspended in 0.5 mL of 1M acetic acid containing 10 μg/mL pepstatin A. ET-1 concentration of the supernatant was analyzed (detailed below) and normalized to the protein content of the supernatant and the precipitate.
4) *ET-1 measurement.* Human Endothelin-1 QuantiGlo ELISA Kit (QET00B; R&D Systems, Minneapolis, MN) was used to measure tissue-extracted ET-1 peptide content and urinary ET-1 level according to manufacturer’s protocol. Samples from both genotypes and sexes were randomly aliquoted onto the microplate. Luminescence signal was measured using a microplate reader (Synergy HA; Agilent/BioTek, Santa Clara, CA).
5) *Bradford protein assay.* Bradford protein assays were used to measure protein concentration in the supernatant and precipitate after ET-1 extraction from renal tissues. Bovine serum albumin (J60205. AC; Thermo Fisher Scientific) was used to prepare standards by dilution in phosphate-buffered saline (PBS) (J62036. K3; Thermo Fisher Scientific). Samples and standards were aliquoted onto 96-well plate, and Quick Start Protein Dye (5,000,205; Quick Start) was added to every well. After 10 min of incubation at room temperature, colorimetry signal was measured using microplate reader (Synergy HA; Agilent/BioTek) at 595 nm wavelength.
6) *Real time PCR.* RNA from renal cortical, outer medullary, and inner medullary tissues was isolated using Purelink Mini RNA kit (12–183-018A; Thermo Fisher Scientific). The isolated RNA was treated with DNA removal Kit (AM 1906; Thermo Fisher Scientific) and reverse transcribed using iScript cDNA Synthesis Kit (1,708,891; Bio-Rad, Hercules, CA). The resulting cDNA was quantified to mRNA by real time iTaq universal probes (1,725,131; Bio-Rad) using TaqMan (Thermo Fisher Scientific) primer gene expression assay for ET-1 (Mm00438656_m1), ET_A_ (Mm01243722_m1), ET_B_ (Mm00432989_m1), and GAPDH (Mm99999915_g1) primers. Gene expression was quantified relative to GAPDH using 2^−ΔΔCT^ method mentioned previously (Boesen et al., 2008).

### ET-1 immunostaining

Kidney tissues were fixed in 4% paraformaldehyde (032–059; Thermo Fisher Scientific) for 24 h at room temperature on a shaker, transferred to 70% ethanol (04,355,223, Thermo Fisher Scientific) for 24 h, then processed for paraffin embedding by the UAB Pathology Core Laboratory. Tissues were cut longitudinally into 5-μm thick sections. Kidney sections were stained with ET-1 antibody (H-023–01; Phoenix Pharmaceuticals, Burlingame, CA) and horseradish peroxidase-linked secondary antibody (7074P2; Cell Signaling Technology, Danvers, MA) using the manufacturer’s instructions. Briefly, tissues sections were subjected to antigen retrieval at high temperatures (120°C) and high pressure in citrate buffer (pH 6.0) using a pressure cooker. Sections were incubated with Dulbecco’s PBS (14,190–144; Thermo Fisher Scientific) containing 10% goat serum (005–000-121; Jackson ImmunoResearch Laboratories, West Grove, PA). Sections were then incubated with the primary antibody for ET-1 overnight, followed by the appropriate secondary antibody, listed above. Stained kidney sections were scanned by the Nikon Tie2 eclipse microscope. Immunohistochemistry images were analyzed using ImageJ Fiji software (ver. 1.53q), as previously detailed (Crowe and Yue, 2019), to quantify ET-1 staining, as percent area, in the renal cortex.

### Statistical methods

Statistical analysis was performed using two-way *ANOVA* and Šídák’s multiple comparisons *post hoc* tests. All data are presented as means ± SE. The specific statistical analysis test used for each data set is specified in each figure and table legend. An alpha level of 0.05 was used for determining statistical significance. Statistical analysis was performed using GraphPad Prism software (ver. 9). Some urine samples collected from metabolic cage studies did not yield adequate volume for ET-1 assays, therefore not all urine samples were analyzed for ET-1 level.

## Results

**FIGURE 2:**
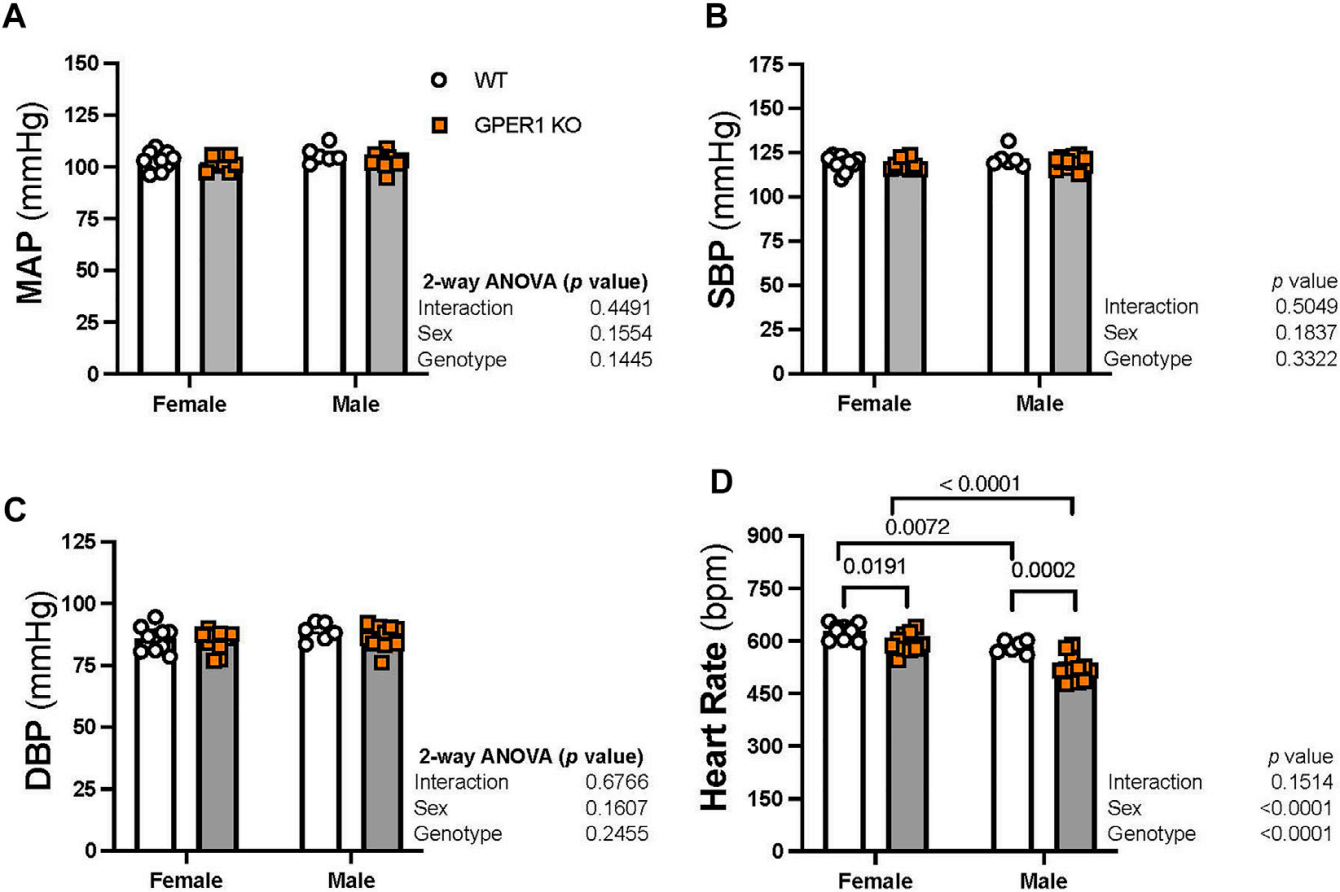
Deletion of GPER1 did not affect blood pressure. 24 h mean arterial blood pressure (MAP, panel **(A)**, systolic blood pressure (SBP, panel **(B)**, diastolic blood pressure (DBP, panel **(C)**, and heart rate (panel **(D)**) were recorded in female and male GPER1 KO and WT mice. Data represents mean ± SEM (females: WT: *n* = 10, GPER1 KO: *n* = 10; males: WT: *n* = 6, GPER1 KO: *n* = 12). Statistical comparisons were performed by 2-way ANOVA with Šídák’s *post hoc* test for multiple comparisons. ANOVA results are listed next to each figure panel, and *p* values < 0.05 for *post hoc* tests are displayed on the figures. Abbreviations: bpm, beats per minute; DBP, diastolic blood pressure; ET-1, endothelin-1; GPER1 KO, G protein-coupled estrogen receptor 1 knockout; MAP, mean arterial pressure; SBP, systolic blood pressure; SE, standard error; WT, wildtype.

*Blood pressure.* No significant sex or genotypic differences were observed in MAP, systolic blood pressure, and diastolic blood pressure (Figures 2A–C). GPER1 deletion decreased heart rate (HR) in both female and male mice (Figure 2D). Female GPER1 KO and WT mice had higher HR compared to genotype-matched males (Figure 2D).

*Metabolic cage parameters.* No genotypic differences were evident in data obtained using metabolic cages (Table 1). GPER1 KO mice weighed slightly less than WT littermates, but this body weight difference did not reach statistical significance (*p* = 0.0641). Male mice had higher body weight, food intake, water intake, urine flow, and urinary Na^+^ excretion compared to females. The sex difference observed in water intake was genotype-specific; male GPER1 KO mice consumed more water than female GPER1 KO mice. No significant differences in urinary K^+^ excretion, plasma [Na^+^] or [K^+^] were observed between the groups (Table 1).

**TABLE 1:** Metabolic cage parameters.

	Female WT	Female KO	Male WT	Male KO	2-Way ANOVA (p)
Body weight (g)	21.36 ± 0.32	20.71 ± 0.58	30.11 ± 0.70****	28.19 ± 0.88****	p_interaction_ = 0.3564
					p_sex_ < 0.0001
					p_genotype_ = 0.0641
Food intake (g/24 h)	3.81 ± 0.08	3.93 ± 0.09	4.26 ± 0.13*	4.43 ± 0.16*	p_interaction_ = 0.8583
					p_sex_ = 0.0005
					p_genotype_ = 0.2525
Water intake (mL/24 h)	3.60 ± 0.13	3.43 ± 0.17	3.80 ± 0.20	4.18 ± 0.22*	p_interaction_ = 0.1523
					p_sex_ = 0.0158
					p_genotype_ = 0.5786
Urine flow (mL/24 h)	0.26 ± 0.06	0.34 ± 0.06	0.58 ± 0.09	0.59 ± 0.14	p_interaction_ = 0.7334
					p_sex_ = 0.0089
					p_genotype_ = 0.6344
Urinary Na^+^ excretion (mEq/24 h)	0.091 ± 0.026	0.147 ± 0.024	0.171 ± 0.012	0.186 ± 0.034	p_interaction_ = 0.4457
					p_sex_ = 0.0351
					p_genotype_ = 0.1981
Urinary K^+^ excretion (mEq/24 h)	6.60 ± 1.82	9.89 ± 1.56	9.77 ± 0.71	11.33 ± 2.01	p_interaction_ = 0.6165
					p_sex_ = 0.1882
					p_genotype_ = 0.1665
Urinary aldosterone excretion (pg/24 h)	306.79 ± 81.45	315.98 ± 73.13	396.06 ± 43.95	410.56 ± 49.66	p_interaction_ = 0.9669
					p_sex_ = 0.1596
					p_genotype_ = 0.8536
Plasma [Na^+^] (mEq/L)	159.35 ± 2.87	157.84 ± 1.85	165.77 ± 3.26	164.01 ± 5.23	p_interaction_ = 0.9735
					p_sex_ = 0.0832
					p_genotype_ = 0.6457
Plasma [K^+^] (mEq/L)	3.71 ± 0.05	3.73 ± 0.10	3.45 ± 0.15	3.67 ± 0.05	p_interaction_ = 0.2952
					p_sex_ = 0.1078
					p_genotype_ = 0.2123

Data represents mean ± SE (for urine electrolyte data: females: WT: *n* = 12, GPER1 KO: *n* = 8; males: WT: *n* = 10, GPER1 KO: *n* = 12; for aldosterone data: all groups: *n* = 7–8/group; for plasma samples: all groups: *n* = 9/group). 2-way ANOVA, test was performed for statistical comparisons, and multiple comparisons were performed by Šídák’s *post hoc* tests. 2-way ANOVA test *p* values are shown on the far-right column and asterisks represent *p* values < 0.05 for *post hoc* test. Abbreviations: KO, G protein-coupled estrogen receptor 1 knockout; SE, standard error; WT, wildtype, *p ≤ 0.05 vs corresponding female genotype and ****p ≤ 0.0001 vs corresponding female genotype.

**FIGURE 3:**
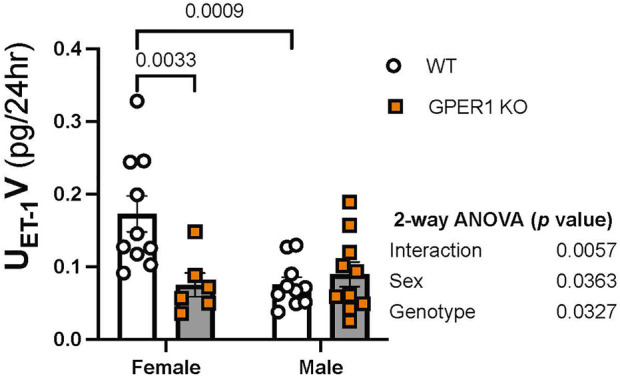
GPER1 deletion eliminates the sex difference in ET-1 excretion. Urinary ET-1 excretion rate (U_ET-1_V) was measured in female and male GPER1 KO and WT mice. Data represents mean ± SE (female GPER1 KO: *n* = 6, all other groups: *n* = 10/group). Statistical comparisons and multiple comparisons were performed by 2-way ANOVA and Šídák’s *post hoc* test, respectively. ANOVA results are listed beside each figure panel, and *p* values for *post hoc* tests are displayed on the figures. Abbreviations: GPER1 KO, G protein-coupled estrogen receptor 1 knockout; SE, standard error; U_ET-1_V, endothelin-1 urinary excretion rate; WT, wildtype.

*Renal ET-1 signaling system.* To determine the effects of GPER1 deletion on the renal ET-1 system, we first determined the urinary excretion rate of ET-1 (Figure 3). Our results revealed sex and genotypic differences in urinary ET-1 excretion (Figure 3). Among WT mice, females excreted significantly more ET-1 compared to males (Figure 3), which is consistent with recent reports in rats and humans (Gohar et al., 2022). Interestingly, female GPER1 KO mice excreted significantly lower levels of urinary ET-1 compared to female WT littermates (Figure 3), suggesting that GPER1 contributes to the sex difference in urinary ET-1 excretion.

**FIGURE 4:**
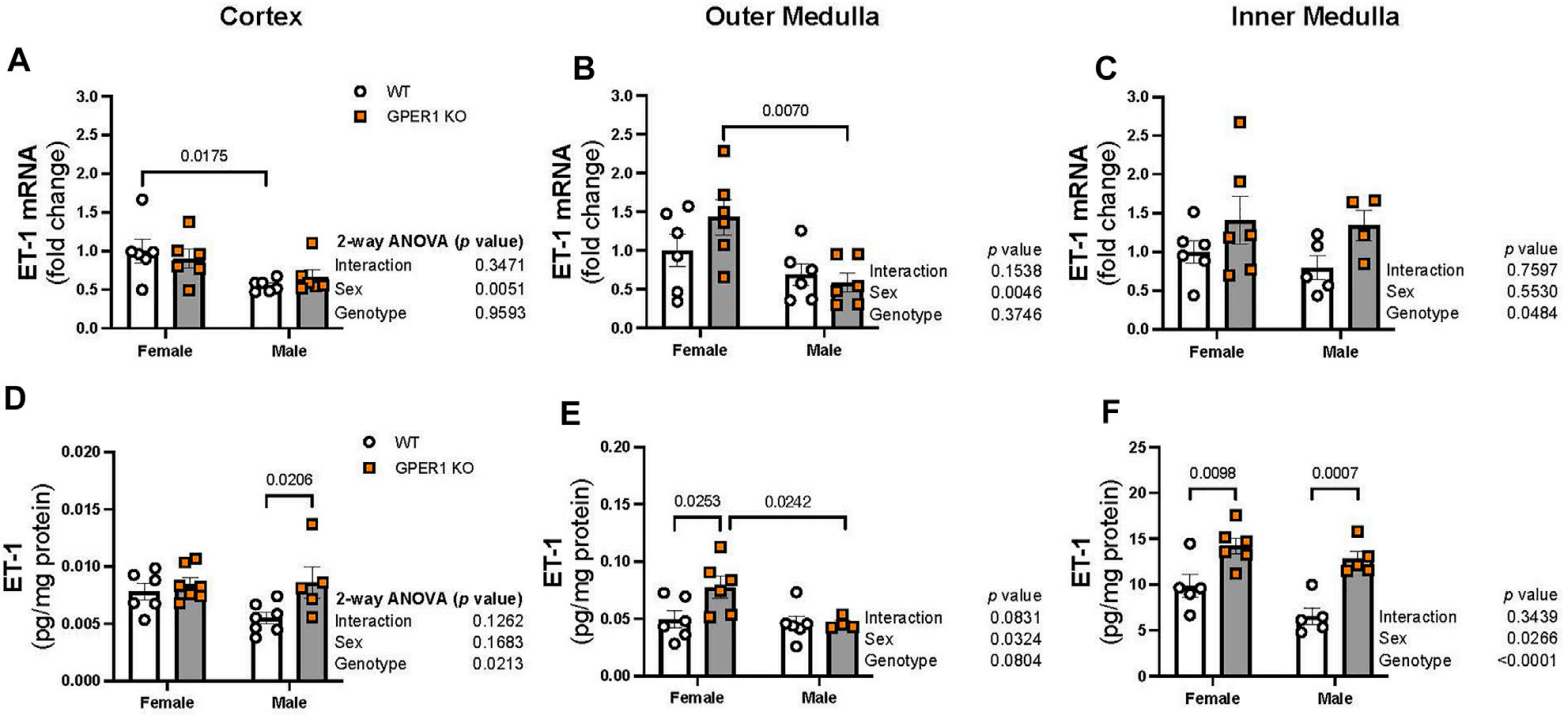
GPER1 deletion increases ET-1 protein content within the renal cortex and medulla in a sex-specific manner. ET-1 mRNA expression (panels **(A–C)**) and protein content (panels **(D–F)**) were measured in the renal cortex, outer, and inner medulla tissues obtained from female and male GPER1 KO and WT mice. ET-1 mRNA fold change was calculated relative to female WT group. ET-1 tissue content was calculated by measuring ET-1 and protein concentration of tissue homogenates. Data represents mean ± SE (females: WT: *n* = 5–6/group, GPER1 KO: *n* = 6–7/group; males: WT: *n* = 5–7/group, GPER1 KO: *n* = 4–6/group). *n* value varies among kidney sections. Statistical comparisons were performed by 2-way ANOVA as presented beside each figure. Multiple comparisons were performed by Šídák’s *post hoc* test and presented on the graphs. Abbreviations: ET-1, endothelin-1; GPER1 KO, G protein-coupled estrogen receptor 1 knockout; SE, standard error; WT, wildtype.

Figure 4 displays ET-1 mRNA expression and peptide content measured in the renal cortex, outer, and inner medulla regions. In the renal cortex and outer medulla, females showed enhanced ET-1 mRNA expression compared to male littermates (Figures 4A, B). In particular, cortical and outer medullary tissues from female WT and GPER1 KO mice, respectively, expressed higher ET-1 mRNA compared to corresponding genotype-matched male groups (Figures 4A, B). However, the inner medullary region displayed a genotypic difference, rather than a sex-difference, in ET-1 mRNA expression (Figure 4C). Specifically, GPER1 KO mice expressed more inner medullary ET-1 mRNA than WT mice (Figure 4C).

Among all measured kidney regions, the highest level of ET-1 peptide content was evident in the inner medullary tissues (Figures 4D–F), similar to previous observations (Kitamura et al., 1989; Kohan and Padilla, 1992). GPER1 deletion increased ET-1 peptide content in different regions of the kidney in a sex-specific manner (Figures 4D–F). Cortical ET-1 peptide content was greater in male GPER1 KO mice compared to male WT mice (Figure 4D). However, GPER1 deletion significantly increased outer medullary ET-1 peptide content in females but not males (Figure 4E). Within the inner medullary tissues, GPER1 deletion remarkably amplified ET-1 peptide content in both sexes (Figure 4F).

**FIGURE 5:**
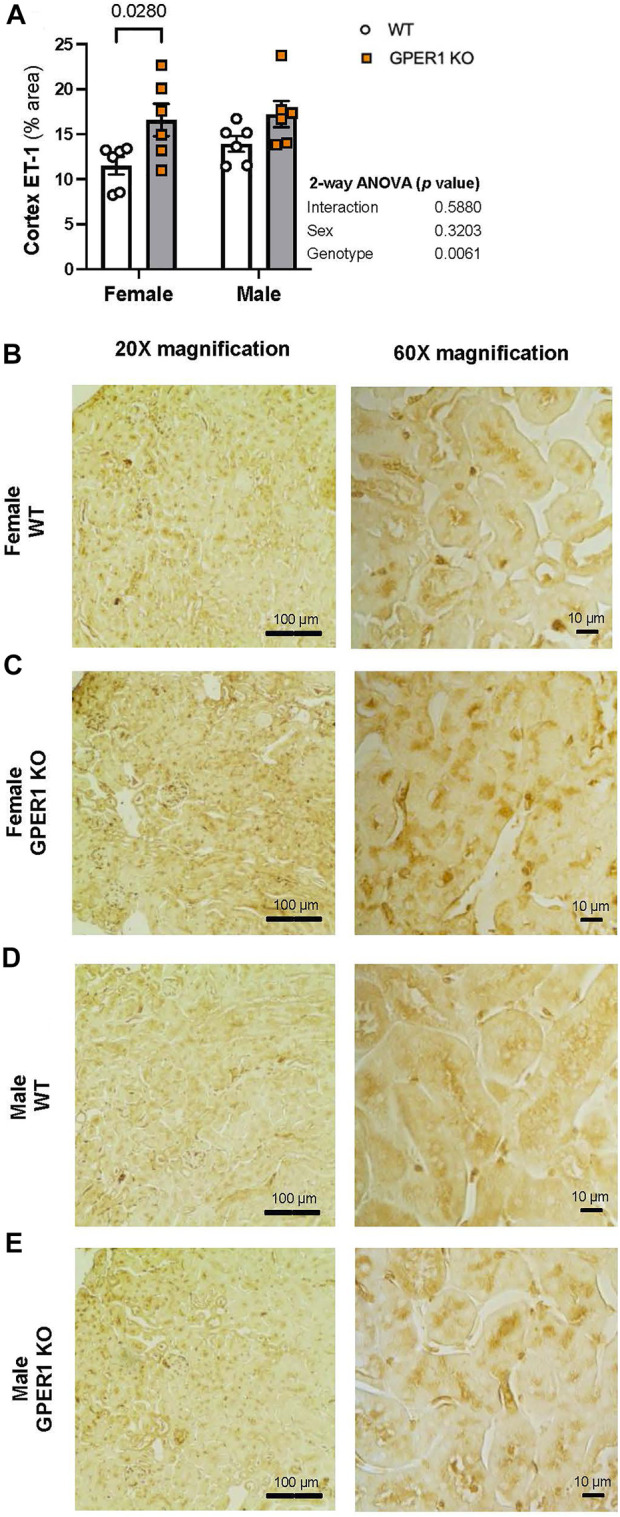
GPER1 deletion increases ET-1 immunohistology staining within the renal cortex. Renal ET-1 was assessed by ET-1 antibody immunohistology staining and ET-1 percent area stained was calculated by image analysis in renal cortex (panel **(A)**) of female and male GPER1 KO and WT mice tissues. Panels **(B–E)** represent images from renal cortex ET-1 staining at ×20 (left column) and ×60 (right column) magnification. Data represents mean ± SE (all groups: 6/group). Statistical comparisons were performed by 2-way ANOVA as presented beside each figure. Multiple comparisons were performed by Šídák’s *post hoc* test and presented on the graphs. Abbreviations: ET-1, endothelin-1; GPER1 KO, G protein-coupled estrogen receptor 1 knockout; SE, standard error; WT, wildtype. Scale bar: 100 μm (left), 10 μm (right); All images were increased by 20% in brightness and 20% in contrast.

Immunohistology staining for ET-1 in kidney sections revealed genotype-related differences in cortical ET-1 expression pattern (Figure 5). Higher contrast areas within the image mark the stained ET-1 peptide. No sex-related differences were evident in renal ET-1 expression pattern. The percentage of renal cortical area stained with ET-1 was greater in female GPER1 KO mice compared to corresponding WT mice (Figure 5B). In female GPER1 KO mice, stronger ET-1 staining was observed in the cytoplasm of the proximal tubules, suggesting that GPER1 deletion in female mice increases ET-1 storage in renal tubular cells, restricting its release in urine.

**FIGURE 6:**
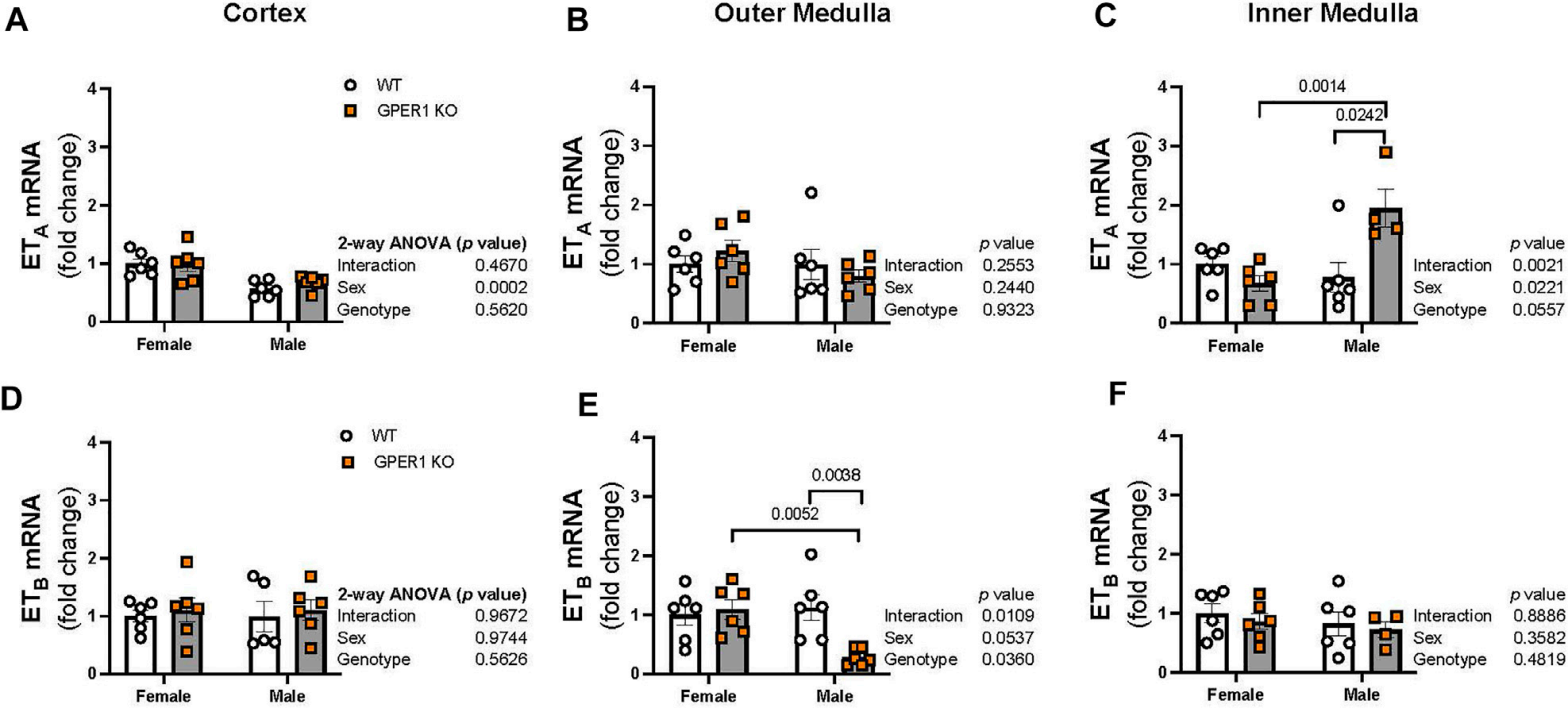
GPER1 deletion regulates renal medullary ET_A_ and ET_B_ expression in a sex-specific manner. mRNA expression of ET_A_ **(A–C)** and ET_B_ **(D–F)** were measured in the renal cortex, outer, and inner medullary tissues of female and male GPER1 KO and WT mice. ET_A_ and ET_B_ mRNA expression is calculated relative to female WT group. Data represents mean ± SE (females: WT: n = 6/group, GPER1 KO: n = 6/group; males: WT: n = 5–6/group, GPER1 KO: n = 4–6/group). Statistical comparisons were performed by 2-way ANOVA. Multiple comparisons were performed by Šídák’s *post hoc* test. ANOVA results are listed next to each figure panel, and *p* values < 0.05 for *post hoc* tests are displayed on the figures. Abbreviations: ET-1, endothelin-1; ET_A_, endothelin-1 receptor A; ET_B_, endothelin-1 receptor B; GPER1 KO, G protein-coupled estrogen receptor 1 knockout; SE, standard error; WT, wildtype.

ET-1 actions are mediated *via* activation of its downstream signaling G protein-coupled receptors, ET_A_ and ET_B_, which were measured in each kidney section. In the renal cortex, ET_A_ mRNA expression was higher in females compared to males (Figure 6A). No sex or genotype differences in outer medullary ET_A_ mRNA expression were observed (Figure 6B). Within the inner medulla, GPER1 deletion significantly increased ET_A_ mRNA expression in male, but not female, mice (Figure 6C). No significant sex or genotypic differences were observed for ET_B_ mRNA expression within the cortex or inner medulla (Figures 6D, F). However, GPER1 deletion decreased outer medullary ET_B_ mRNA expression in male, but not female, mice (Figure 6E).

## Discussion

An understanding of the molecular mechanisms underlying the protection of premenopausal women against hypertension is foundational to the development of future therapeutic targets. Renal ET-1 signaling plays a critical role in the maintenance of Na^+^ homeostasis and consequently blood pressure. The current study demonstrates that GPER1 promotes urinary ET-1 excretion in females, lowers renal ET-1 peptide and mRNA expression levels in a sex-specific and region-specific manner in the kidneys, and regulates renal medullary ET receptor expression in males. ET-1 immunostaining results suggest that GPER1 signaling may promote the secretion of intracellular ET-1, as GPER1 deletion caused significantly greater ET-1 storage in proximal tubule cells. Overall, our data identify the novel estrogen receptor, GPER1, as an upstream regulator for renal ET-1 signaling system in both males and females.

Male-female differences in renal ET-1 signaling have been observed in a wide range of studies (Nakano and Pollock, 2009; Jin et al., 2013; Johnston et al., 2016; Gohar et al., 2020; Gohar et al., 2022). Ovary-intact female, but not male or OVX female, rats have been shown to utilize ET_A_ receptors to induce natriuresis while being fed a high salt diet (Nakano and Pollock, 2009). More so, estrogen supplementation prevented the OVX-induced increase in inner medullary ET receptor expression in rats (Gohar et al., 2016b), indicating that sex steroids regulate the renal ET-1 signaling system. Similarly, the current findings demonstrate sex differences in ET-1 mRNA expression and ET-1 protein content in the kidney. In cortical and outer medullary tissues, female GPER1 KO and WT mice have greater ET-1 mRNA expression compared to males. In outer and inner medullary tissues, female mice regardless of their genotype have increased ET-1 peptide content than males. These results support previous evidence (Nakano and Pollock, 2009; Jin et al., 2013; Johnston et al., 2016; Gohar et al., 2020; Gohar et al., 2022) that females have greater ET-1 activity than males. Furthermore, our results reveal that GPER1 deletion increases outer medullary ET-1 mRNA expression and ET-1 protein content in females but not males. This suggests that GPER1 has a female-specific role in downregulating renal ET-1 content in the outer medulla.

The current study suggests that GPER1 activity downregulates renal inner medullary ET-1 content in both sexes, since GPER1 deletion results in greater inner medullary ET-1 mRNA expression and ET-1 peptide content in male and female GPER1 KO mice. Of note, the IMCD produces the highest levels of renal ET-1 (Kitamura et al., 1989). Factors that increase inner medullary ET-1 production include tubular flow/shear (Lyon-Roberts et al., 2011), extracellular fluid volume expansion (Kohan and Padilla, 1993), and increased dietary salt intake (Lyon-Roberts et al., 2011). In addition, IMCD ET-1 production is required for preventing salt-dependent hypertension (Ahn et al., 2004). Specifically, Ahn et al. showed that collecting duct ET-1 knock out mice elicit hypertension, highlighting a crucial contribution for the renal tubular ET-1 signaling as a pro-natriuretic factor (Ahn et al., 2004). No genotypic differences in water intake, urinary flow, Na^+^ excretion, or plasma Na^+^ were demonstrated in the current study, pointing to GPER1 as an independent regulator for IMDC ET-1 production. Given that ET-1 mRNA expression is lower in the inner medulla of male and female GPER1 KO, we speculate that GPER1 signaling reduces renal ET-1 transcription possibly by deactivating certain ET-1 transcription factors. Furthermore, we speculate that the increased renal ET-1 peptide content in GPER1 KO mice may be due to GPER1 involvement in the release of ET-1 storage vesicles. Reports of vesicles storing ET-1 and its precursors have been located in endothelial cells of coronary arteries (Russell et al., 1998a; Russell et al., 1998b), but evidence for such storage machineries in renal tissues remains to be determined.

Our findings suggest that GPER1 has a sex-specific influence on water intake, since male GPER1 KO mice drank more water than female GPER1 KO mice, and this sex-difference was not apparent among WT mice. Non-etheless, previous evidence shows that GPER1 activation reduces water intake in OVX female rats (Santollo and Daniels, 2015). In fact, GPER1 is expressed in the paraventricular and supraoptic nuclei of the hypothalamus in similar levels between male and female rats (Brailoiu et al., 2007; Sakamoto et al., 2007). Of note, both nuclei are important structures for fluid homeostasis (Brailoiu et al., 2007; Sakamoto et al., 2007). Since thirst mechanisms are also influential for blood pressure regulation, further investigation is needed to understand the sex-specific influence of GPER1 signaling on thirst.

Our results suggest that GPER1 is required for greater female urinary ET-1 excretion. The present study shows that urinary ET-1 excretion is greater in female WT mice, compared to males. Similarly, we have recently demonstrated a higher capacity of females to excrete ET-1, compared to males, in rats and humans maintained on a Na^+^-controlled diet (Gohar et al., 2022). Importantly, it has been demonstrated that urinary ET-1 excretion is diminished in human subjects with essential and salt sensitive hypertension, compared to non-hypertensive subjects (Hoffman et al., 1994). Thus, the greater ET-1 excretion in female mice, rats and humans may be a contributing factor in the female protection against hypertension. Notably, the current study demonstrated that this sex-difference in ET-1 excretion was eliminated by GPER1 deletion indicating that GPER1 is necessary for the increased urinary ET-1 excretion in females. As previously mentioned, our results show that GPER1 deletion increases inner medullary ET-1 mRNA expression and peptide content. This discrepancy in renal ET-1 tissue content and urinary ET-1 excretion suggests that GPER1 regulates ET-1 storage and/or degradation in the kidney. It is well established that ET_B_ receptors facilitate ET-1 clearance by sequestering and binding ET-1 for degradation (Fukuroda et al., 1994; Dupuis et al., 1996). Additional molecular studies are needed to clarify the contribution of GPER1 signaling to ET-1 clearance by ET_B_ receptors.

The current study indicates that GPER1 signaling regulates renal ET-1 production in a sex-specific and a kidney region-specific manner. Of note, ANOVA results of ET-1 peptide content measurements and the immunohistochemistry analysis determined genotype as a significant factor for cortical ET-1 peptide levels. However, this genotype effect was more pronounced in males for the ET-1 peptide and more pronounced in females for the ET-1 staining analysis. Additionally, our previous study showed that GPER1 deletion decreases ET-1 mRNA expression in female, but not male, kidney tissues (Gohar et al., 2020). It is important to note in the previous study, we only investigated ET-1 and ET receptor expression in whole kidney homogenates, and we did not attempt to quantify region-specific changes. In addition, the current study utilized relatively younger mice (12–16 weeks-old), compared to our previous study (Gohar et al., 2020) that utilized 18–25 weeks-old GPER1 KO mice. Importantly, it has been demonstrated that renal GPER1 expression is upregulated by age in female kidneys (Gurrala et al., 2021). Further studies are needed to determine the impact of age on the crosstalk between renal GPER1 and ET-1 signaling systems.

The present findings reveal a drastic, male-specific increase in inner medullary ET_A_ mRNA expression and decrease in outer medullary ET_B_ mRNA expression in response to GPER1 deletion. Decreased renal ET_B_ in male GPER1 KO kidneys has a negative impact on the natriuretic capacity of the renal ET-1 system, as ET_B_ is the primary facilitator of ET-1-induced natriuresis (Gariepy et al., 2000; Hoffman et al., 2000; Nakano et al., 2008). Although we did not observe differences in urinary Na^+^ excretion and blood pressure in response to GPER1 deletion, this likely reflects the maintenance of overall fluid-electrolyte balance that would be expected in a steady state. Further studies are required to determine whether salt loading could uncover any male-specific deficiency in the natriuretic capacity of the GPER1 KO kidneys.

Multiple lines of evidence show that GPER1 activation lowers blood pressure acutely in OVX SD rats (Gohar et al., 2020) and chronically in OVX mRen2. Lewis rats (Lindsey et al., 2009), along with enhanced endothelium-dependent vasodilation in OVX mRen2. Lewis rats (Lindsey et al., 2011) and male and female SD rats (Broughton et al., 2010). Additionally, previous evidence points to a crosstalk between estrogen signaling and the renin-angiotensin-aldosterone system (Xue et al., 2007; Lindsey et al., 2009; Cheng et al., 2019) and nitric oxide production (Rubanyi et al., 1997; Chen et al., 1999; Zhu et al., 2002; Muller-Delp et al., 2003), separately, for modulating blood pressure. However, urinary aldosterone levels in the GPER1 KO mice were not affected. The renal ET-1 system sodium handling function contributes to the circadian control of blood pressure (Crislip et al., 2018; Douma et al., 2021). Specifically, during acute salt loading, natriuresis is delayed in male ET_B_ deficient rats compared to female ET_B_ deficient rats and rats with the receptor (Johnston et al., 2016). The current findings did not show any significant differences in blood pressure in response to GPER1 deletion in mice of either sex. Similarly, Mårtensson et al. reported no blood pressure differences in male and female GPER1 KO mice, compared to WT mice at 6 months of age (Mårtensson et al., 2009). Whether a compensatory upregulation in estrogen receptor α (ERα) and estrogen receptor β (ERβ) signaling in response to GPER1 deletion contributes to the lack of a blood pressure phenotype in the GPER1 KO mice remains to be determined. Similarly, it is undetermined whether another sodium-handling mechanism compensated during nephrogenesis in GPER1 KO mice development to account for the lack of differences in urinary sodium excretion. Importantly, it was reported that 9-month-old male and female GPER1 KO mice elicit an increase in blood pressure (Mårtensson et al., 2009), indicating an important role for GPER1 in the maintenance of blood pressure in aged, compared to young, mice. Our lab has recently reviewed and outlined supporting evidence for GPER1 as a novel, therapeutic target for hypertension, especially in postmenopausal women (Gohar, 2020).

The current study showed that GPER1 deletion reduces HR in both male and female mice which is consistent with our previous findings of systemic G1 administration increasing HR in OVX rats (Gohar et al., 2020). In addition, Romano et al. (Romano et al., 2017) demonstrated that GPER1 mutant zebrafish embryos had reduced HR due to GPER1 increasing triiodothyronine thyroid hormone concentrations (Romano et al., 2017). However, this was only shown in zebrafish embryos and later development may impact GPER1 influence on thyroid hormone levels. Currently, there is contradictory evidence regarding the impact of GPER1 on the regulation of HR. Of note, the current study and Romano et al. (Romano et al., 2017) directly measured HR in whole animal models. Reports that indicate GPER1 reduction in HR predominantly used *in vitro* models (Ullrich et al., 2008; Whitcomb et al., 2020). Specifically, estrogen administration in isolated myocytes from ERα KO and ERβ KO mice (Ullrich et al., 2008) and activation of GPER1 in isolated mouse myocytes (Whitcomb et al., 2020) have been shown to inhibit L-type Ca^2+^ channel potentiation. Additional studies are needed to investigate the contribution of cardiac vs extracardiac GPER1 in the regulation of HR.

Overall, our findings demonstrate that deletion of GPER1 increases ET-1 mRNA expression and peptide levels in a region and sex-specific manner. Most notably, our data indicates that GPER1 is required for greater urinary ET-1 excretion in females and that GPER1 downregulates inner medullary ET-1 content in both sexes. GPER1 deletion in males increased inner medullary ET_A_ and decreased outer medullary ET_B_ mRNA expression, suggesting that GPER1 may be responsible for the physiological balance of renal ET receptors required for proper ET-1-dependent Na^+^ handling in males. Thus, we provide evidence for GPER1 signaling as an upstream regulator for the renal ET-1 system in both sexes.

## Data Availability

The original contributions presented in the study are included in the article/Supplementary Material, further inquiries can be directed to the corresponding author.

## Footnotes

1 EG is also affiliated with the Department of Pharmacology and Toxicology, Faculty of Pharmacy, Alexandria University, Egypt.
